# Health-related quality of life in men with localized prostate cancer treated with radiotherapy: validation of an abbreviated version of the Expanded Prostate Cancer Index Composite for Clinical Practice in Spain

**DOI:** 10.1186/s12955-021-01856-z

**Published:** 2021-09-25

**Authors:** Almudena Zapatero, Xavier Maldonado Pijoan, Antonio Gómez-Caamaño, José Pardo Masferrer, Víctor Macías Hernández, Asunción Hervás Morón, Julia Luisa Muñoz García, Amalia Palacios Eito, Paloma Anguita-Alonso, Cristina González-Junco, José López Torrecilla

**Affiliations:** 1 Department of Radiation Oncology, Hospital Universitario de La Princesa, Madrid, Spain; 2 Department of Radiation Oncology, Hospital Universitari Vall d’Hebron, Barcelona, Spain; 3 Department of Radiation Oncology, Hospital Clínico Universitario de Santiago de Compostela, Santiago de Compostela, A Coruña, Spain; 4 Department of Radiation Oncology, Hospital Universitari Son Espases, Palma de Mallorca, Spain; 5 Hospital Universitario Y Politecnico La Fe, Valencia, Spain; 6 Department of Radiation Oncology, Hospital Ramon Y Cajal, Madrid, Spain; 7 Department of Radiation Oncology, Hospital Infanta Cristina de Badajoz, Badajoz, Spain; 8 Department of Radiation Oncology, Hospital Universitario Reina Sofía, Córdoba, Spain; 9 Astellas Pharma Inc., Madrid, Spain; 10 Department of Radiation Oncology, ERESA, Hospital General Universitario de València, València, Spain

**Keywords:** Prostate cancer, EPIC, Quality-of-life assessment, Radiotherapy, Brachytherapy

## Abstract

**Background:**

Health-related quality of life (HRQoL) is greatly affected by prostate cancer (PCa) and associated treatments. This study aimed to measure the impact of radiotherapy on HRQoL and to further validate the Spanish version of the 16-item Expanded Prostate Cancer Index Composite (EPIC-16) in routine clinical practice.

**Methods:**

An observational, non-interventional, multicenter study was conducted in Spain with localized PCa patients initiating treatment with external beam radiotherapy (EBRT) or brachytherapy (BQT). Changes from baseline in EPIC-16, University of California-Los Angeles Prostate Cancer Index (UCLA-PCI), and patient-perceived health status were longitudinally assessed at end of radiotherapy (V2) and 90 days thereafter (V3). Psychometric evaluations of the Spanish EPIC-16 were conducted.

**Results:**

Of 516 patients enrolled, 495 were included in the analysis (EBRT, *n* = 361; BQT, *n* = 134). At baseline, mean (standard deviation [SD]) EPIC-16 global scores were 11.9 (7.5) and 10.3 (7.7) for EBRT and BQT patients, respectively; scores increased, i.e., HRQoL worsened, from baseline, by mean (SD) of 6.8 (7.6) at V2 and 2.4 (7.4) at V3 for EBRT and 4.2 (7.6) and 3.9 (8.2) for BQT patients. Changes in Spanish EPIC-16 domains correlated well with urinary, bowel, and sexual UCLA-PCI domains. EPIC-16 showed good internal consistency (Cronbach’s alpha = .84), reliability, and construct validity.

**Conclusion:**

The Spanish EPIC-16 questionnaire demonstrated sensitivity, strong discriminative properties and reliability, and validity for use in clinical practice. EPIC-16 scores worsened after radiotherapy in different HRQoL domains; however, a strong tendency towards recovery was seen at the 3-month follow-up visit.

**Supplementary Information:**

The online version contains supplementary material available at 10.1186/s12955-021-01856-z.

## Background

In Spain, prostate cancer (PCa) is the most common leading type of cancer in terms of incidence and the third most common cause of cancer death in men, with age-standardized incidence and mortality rates of 104.2 and 13.2 per 100,000 men, respectively, in 2018 [1]. There are various treatment options for localized PCa depending on disease stage, including radical prostatectomy, radiotherapy, such as external beam radiotherapy (EBRT) and brachytherapy (BQT) [2]. A recent study showed that for clinically localized PCa in Spain, the majority of patients analyzed (~ 84%) received treatment, with one-third undergoing radiotherapy; ~ 86% were treated with intensity-modulated radiation therapy or 3D radiotherapy and ~ 39% received BQT [3]. However, these therapeutic interventions can negatively impact patients in terms of physical and emotional symptoms [4]. It is also true that the effect on HRQoL strongly depends on the treatment being given, as well as the temporary HRQoL changes. Therefore, HRQoL and functional performance are of paramount importance, as they can provide information on patients’ prognosis and their capacity to tolerate cancer treatment. Patient-reported outcome (PRO) measures are increasingly being used to assess long-term health-related quality of life (HRQoL) [5]. A well-established instrument that is frequently used to assess a patient’s post-intervention-related HRQoL is the 50-item Expanded Prostate Cancer Index Composite (EPIC) questionnaire, which was developed based on the University of California-Los Angeles Prostate Cancer Index (UCLA-PCI) [6, 7]. However, these instruments were mostly designed for use in the research field and they are too lengthy and time consuming to be used in clinical practice, which limits the ability of physicians who treat PCa to accurately assess HRQoL and optimally individualize treatment-related decisions. There are two shorter versions of the EPIC-50 questionnaire, the 26-item (EPIC-26) [8] and the 16-item EPIC for Clinical Practice (EPIC-CP) [9] questionnaires; the latter was specifically designed to be administered in routine clinical practice. EPIC-CP is a one-page questionnaire that measures urinary incontinence, urinary irritation, bowel, sexual, and hormonal HRQoL domains in patients with clinically localized PCa to evaluate aspects of therapy that are most bothersome [9]. The EPIC questionnaire has already been validated and translated in various languages, including Spanish for EPIC-50 [10], Italian for EPIC-26 [11] and German for the shortened EPIC-CP [12]. Still, the abbreviated version of EPIC questionnaire needs to be validated for use in the context of routine clinical practice in Spain. This will enable a less time-consuming evaluation of treatment-related HRQoL.

In the present study we aimed to measure the specific impact of radiotherapy on HRQoL in patients with localized PCa and to validate the measurement properties of the Spanish version of the EPIC-CP questionnaire in routine clinical practice in Spain.

## Methods

### Study design and participant selection

This observational, non-interventional, multicenter study was conducted in Spain. Data were collected between January 2016 and September 2017 in 41 radiation oncology departments.

Male patients aged ≥ 40 years with histopathologically confirmed, localized PCa who were initiating EBRT or BQT treatment were included. Patients were required to complete follow-up visit questionnaires. Patients with prior prostatectomy, prior radiotherapy or BQT treatment, or with N1 or metastatic tumor stage were excluded.

The study was approved by the independent ethics committee of participating centers. Patients gave their voluntary informed consent to take part. The decision on treatment was not affected by this study and was solely based on the investigator’s criteria.

### Study assessments and data collection

Patients had three study visits where clinical variables were collected at baseline, i.e., before the beginning of treatment (EBRT or BQT) [visit 1], at the first follow-up visit, i.e., the final EBRT session or 1 month after the first BQT session or seed implantation (visit 2), and a final follow-up visit approximately 3 months after the end of treatment (visit 3). Demographic data of the patient population were also collected at baseline, such as age and descriptive analyses of the patient's clinical situation, including disease duration and severity and the radiotherapy given.

### Study questionnaires

Patients completed the following PRO measures at each study visit, with the investigator ensuring all sections were completed properly; the median application time for questionnaires used in this study was not measured. The 16-item EPIC-CP questionnaire, referred to as EPIC-16 in this manuscript, consists of five PCa HRQoL domains and measures urinary, bowel, sexual, vitality, and hormonal health. These five domains contain three questions each with a Likert numeric response scale (NRS) of 0–12 (best to worst HRQoL) for items 2–10, giving a total NRS of 0–60 (Table 1) [9]. The Spanish version of EPIC-50 by Ferrer, et al. was used to create EPIC-16 used in this study [10].

**Table 1:** EPIC-16 and UCLA-PCI domains and response scales

	Instrument	
	EPIC-16	UCLA-PCI
Domains	Urinary incontinence Urinary irritation/obstruction Bowel function Sexual function Vitality/hormonal function	Urinary function Urinary bother Bowel function Bowel bother Sexual function Sexual bother
NRS range, points		
Domain score Total score	0–12 0–60 (best to worst HRQoL)	0–100 0–100 (worst to best HRQoL)

The UCLA-PCI is a 20-item questionnaire with six domains, with an NRS of 0–100 (worst to best HRQoL) and measures the function and degree of impairment in the urinary, intestinal, and sexual domains (Table 1) [7]. Additionally, patients completed the patient-perceived state of health measure, a one-item questionnaire in which patients evaluated their own general PCa-related health on that day as “Very good”, “Fairly good”, “Slightly good”, “Neither good nor bad”, “Slightly bad”, “Quite bad”, or “Very bad”.

### Psychometric validations of the Spanish version of the EPIC-16 questionnaire

The psychometric properties of the EPIC-16 questionnaire were evaluated for sensitivity, reliability, construct, and longitudinal validity and responsiveness for the total number of patients included in the study at baseline versus the follow-up visits (see Additional file 1). This sample size could be altered due to missing responses and non-valid scores for each domain or subscale.

### Statistical analyses

Descriptive statistics were used to summarize patient demographic characteristics, including age, disease stage, and radiotherapy treatment. The mean, standard deviation, median, minimum, and maximum were used for the description of the continuous variables, and according to the distribution of the variable analyzed, the quartiles were also presented. The number and percentage of patients per response category were used for description of the categorical variables. Estimates were made by points and from the 95% CIs for main result variables.

The questionnaire's validity was analyzed for the total number of patients included in the study in terms of construct validity and longitudinal validity. To evaluate the construct's validity, the scores observed in the EPIC-16 questionnaire were compared using analysis of variance. The questionnaire was expected to discriminate between levels of seriousness and worsening of the disease. The correlation between the questions of the EPIC-16 and those of the UCLA-PCI was also analyzed, as well as the relationship between both scores. In parallel, the scores observed in the EPIC-16 questionnaire were compared to patient-perceived overall health using analysis of variance. To evaluate longitudinal validity, the changes observed in the questionnaire between the baseline visit and the follow-up visit were compared to the changes observed in seriousness, RT group, and the UCLA-PCI questionnaire. The bivariate tests corresponding to the characteristics of the variables analyzed were used for the analysis.

The correlations between outcomes in EPIC-16 and UCLA-PCI domains were tested using Spearman rank correlation coefficients. Responsiveness was assessed using the Student t test for paired data. The questionnaire scores at baseline versus each follow-up visit and scores before and after the type of radiotherapy (EBRT or BQT) were compared using a statistical significance level of 0.05, which was used for all statistical tests performed.

## Results

### Patient demographics and baseline characteristics

**Fig. 1:**
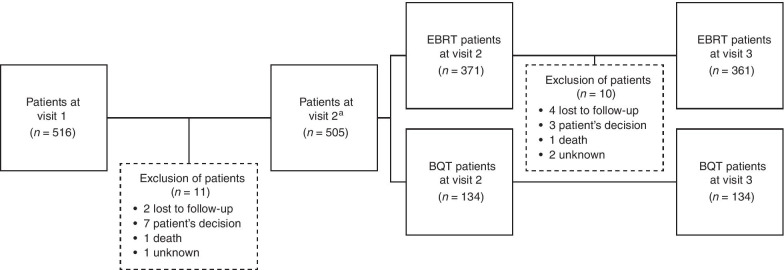
Patient flow chart. ^a^Patients who withdrew at the first visit were not assigned to any group due to the type of radiotherapy treatment status being recorded at the second visit. *Visit 1* Baseline, *Visit 2* End of radiotherapy, *Visit 3* 90 days after the end of radiotherapy

Overall, 516 patients were enrolled in this study at the baseline visit. Following the second visit, 21 (4.1%) patients were excluded. The remaining 495 patients (95.9%) eligible at visit 3 were included in the analyzed patient population (Fig. 1). A total of 361 patients (72.9%) received EBRT and 134 patients (27.1%) received BQT treatment.

Baseline characteristics stratified by EBRT or BQT treatment groups are summarized in Table 2. At baseline, median patient age (range) was 73.0 (48.0–84.0) years and 67.0 (48.0–82.0) years for the for EBRT and BQT groups, respectively. Most patients who received EBRT had a Gleason score of ≥ 7 (73.1%), while most patients who received BQT had a Gleason score of < 7 (84.3%). A higher proportion of patients received neo-adjuvant hormone therapy in the EBRT group (62.0%) than in the BQT group (6.0%) [Table 2].

**Table 2:** Baseline demographics and disease characteristics

Demographics and disease characteristics	Analyzed patients (*n* = 495)	
	EBRT (*n* = 361)	BQT (*n* = 134)
Median age, years (range)	73.0 (48.0–84.0)	67.0 (48.0–82.0)
Gleason score (grouped),^a^ *n* (%)		
Grade group 1 Grade group 2 Grade group 3 Grade group 4 Grade group 5	97 (26.9) 94 (26.0) 74 (20.5) 55 (15.2) 41 (11.4)	113 (84.3) 21 (15.7) 0 0 0
Median prostate-specific antigen at diagnosis, ng/mL (range)	8.1 (1.7–342.0)	5.9 (1.0–15.7)
Median prostate-specific antigen at initial EBRT/BQT, ng/mL (range)	3.5 (.0–100.0)	5.8 (.8–17.2)
Median testosterone at initial EBRT/BQT, ng/dL (range)	*n* = 177 10.0 (.0–913.0)	*n* = 21 291.0 (2.6–619.6)
TNM stage at initial EBRT/BQT, *n* (%)		
T1c T2 T3	104 (28.8) 148 (41.0) 68 (18.8)	91 (67.9) 19 (14.2) 0
Eastern Cooperative Oncology Group performance status at initial EBRT/BQT, *n* (%)	*n* = 341	*n* = 130
0 1 2 Not available	314 (92.1) 23 (6.7) 4 (1.2) 20	125 (96.2) 5 (3.8) 0 4
Type of BQT, *n* (%)		
Low-dose BQT/seed High-dose BQT	NA	118 (88.1) 16 (11.9)
Location of EBRT treatment, *n* (%)		
Prostate Lymph nodes Seminal vesicles	361 (100) 94 (26.0) 280 (77.6)	NA
Type of EBRT treatment,^b^ *n* (%)		
3D CRT IMRT or VMAT	104 (29.1) 254 (70.9)	NA
Neo-adjuvant hormone treatment, *n* (%)		
Prior to EBRT/BQT Initiating EBRT/BQT	224 (62.0) 219 (97.8) 5 (2.2)	8 (6.0) 8 (100.0) –
Previous interventions, *n* (%)		
Yes No Unknown	141 (39.1) 213 (59.0) 7 (1.9)	25 (18.7) 109 (81.3)
Previous surgery, *n* (%)		
TURP	39 (10.8)	8 (6.0)

^a^Grade group 1 = Gleason score ≤ 6; Grade group 2 = Gleason score 3 + 4 = 7; Grade group 3 = Gleason score 4 + 3 = 7; Grade group 4 = Gleason score 8; Grade group 5 = Gleason scores 9 and 10[28]

^b^Of patients who received EBRT treatment of the prostate (*n* = 358)

*3D CRT* Three-dimensional conformal radiation therapy, *IMRT* Intensity-modulated radiation therapy, *NA* Not available, *TNM* Tumor, node, metastasis, *TURP* Transurethral resection of the prostate, *VMAT* Volumetric-modulated arc therapy

At baseline, the total EPIC-16 mean (standard deviation [SD]) scores were 11.9 (7.5) and 10.3 (7.7) for the EBRT and BQT groups, respectively (see Additional file 1: Fig. S1A) and the UCLA-PCI mean (SD) scores ranged from 27.0 (26.8) to 90.1 (21.5) and 42.2 (30.5) to 90.3 (23.0) across the domains for the EBRT and BQT groups, respectively (see Additional file 1: Fig. S1B).

### Impact of radiotherapy on quality of life

EPIC-16 total scores increased by a mean (SD) of 6.8 (7.6) points at visit 2 and by 2.4 (7.4) points at visit 3 for patients in the EBRT group. For patients who received BQT, the scores increased by a mean (SD) of 4.2 (7.6) and 3.9 (8.2) points at visits 2 and 3, respectively.

**Fig. 2:**
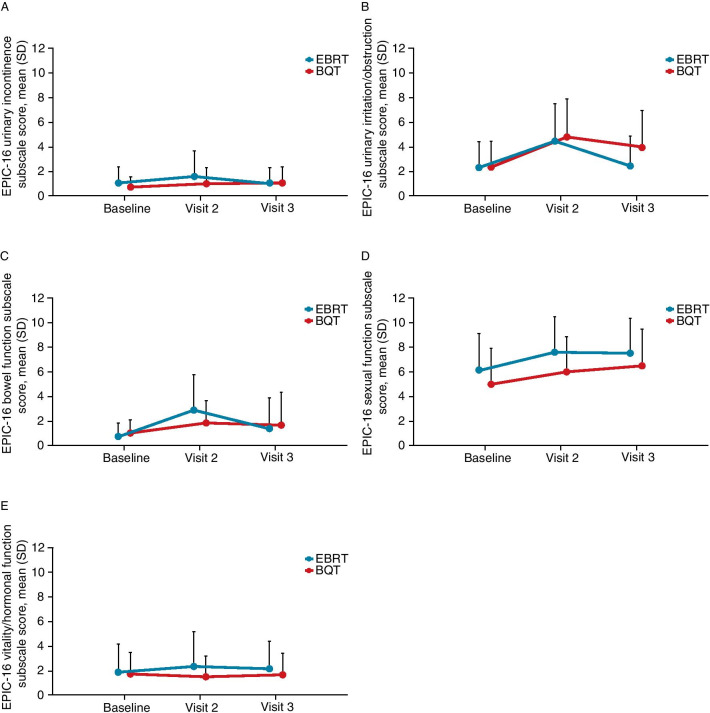
EPIC-16 domain scores for EBRT- and BQT-treated patients. *Visit 2* End of radiotherapy, *Visit 3* 90 days after the end of radiotherapy

Overall, scores increased after radiotherapy, indicating a worsened HRQoL, across all EPIC-16 domains (Fig. 2). At visit 3 particularly, patients recovered their baseline scores in the urinary incontinence, bowel, vitality/hormonal, and urinary irritation domains, with the exception of the bowel and urinary irritation domains in patients receiving BQT (Fig. 2B, C). The sexual domain scores of patients in both groups worsened at both time points and did not recover at visit 3 compared to other domains. Patients who received concomitant hormone therapy, initiated prior to either EBRT or BQT, showed a higher mean score in the sexual domain between study visits 1 and 2 (mean [SD] visit 1: 6.79 [3.16]; mean [SD] visit 2: 8.39 [2.52]) than patients without hormone therapy (mean [SD] visit 1: 4.84 [3.45]; mean [SD] visit 2: 6.15 [3.29]).

For the overall evolution of urinary problems, e.g., item 1 of EPIC-16 (“Overall, how much of a problem has your function been for you?”), responses were similar at baseline for both EBRT and BQT patients (see Additional file 1: Fig. S2). However, more patients treated with EBRT increasingly perceived urinary function as a small-to-big problem at visit 2 (62.0%, *n* = 221) compared with patients treated with BQT (53.2%, *n* = 67). At visit 3, patients’ perceptions returned to baseline, i.e., with urinary condition perceived as less of a problem compared with visit 2 (small-to-big problem: 35.5%, *n* = 126 for EBRT; 48.5%, *n* = 65 for BQT) (see Additional file 1: Fig. S2).

**Fig. 3:**
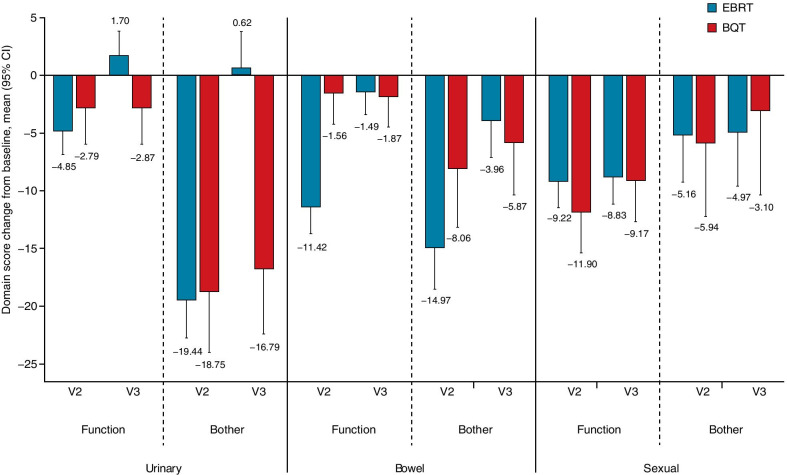
UCLA-PCI domain scores mean change from baseline for EBRT- and BQT-treated patients. *V2* Visit 2 (end of radiotherapy), *V3* Visit 3 (90 days after the end of radiotherapy)

Similarly, for patients who received EBRT, all UCLA-PCI scores showed a decrease at visit 2, indicating worsened HRQoL, especially in urinary bother, bowel function and bother, and sexual function domains (Fig. 3). At visit 3, scores had recovered HRQoL in the urinary function, urinary bother, and bowel function domains. For patients who received BQT, UCLA-PCI scores were decreased at visit 2 in the urinary bother, bowel bother, and sexual function domains, none of which recovered at visit 3 (Fig. 3).

### Validation of the Spanish version of the EPIC-16 questionnaire

Psychometric validation of the EPIC-16 questionnaire was conducted in 484 patients (EBRT, *n* = 357; BQT, *n* = 127).

### Reliability of EPIC-16

A strong internal consistency of the Spanish EPIC-16 questionnaire was demonstrated in the vitality/hormonal (Cronbach’s alpha = 0.729 [95% confidence interval (CI) 0.68–0.77]), urinary incontinence (Cronbach’s alpha = 0.735 [95% CI 0.69–0.77]), urinary irritation (Cronbach’s alpha = 0.777 [95% CI 0.74–0.81]), and bowel function domains (Cronbach’s alpha = 0.879 [95% CI 0.86–0.90]). Only the sexual function domain was < 0.7 (Cronbach’s alpha = 0.616 [95% CI 0.56–0.66]).

Test–retest reliability intraclass correlation coefficient (ICC) was analyzed for 226 patients who did not perceive any change in their health status (according to the patient-perceived state of health measure) after radiotherapy. The ICC was moderate in all domains (range, 0.52–0.66), with the exception of the bowel function domain (ICC = 0.232 [95% CI 0.10–0.36]), indicating that reproducibility in this domain cannot be ensured.

### Sensitivity of EPIC-16

The floor effect, i.e., worst score of 12 for each domain, was present in < 1% of patients, with the exception of the sexual domain for which 24 (4.9%) patients had a maximum score of 12 at baseline, which increased to 44 (9.1%) and 56 (11.5%) patients at visits 2 and 3, respectively (see Additional file 1: Fig. S3).

The ceiling effect, i.e., best score of 0 for each domain, was reached by a high percentage of men in urinary incontinence (*n* = 364; 65.6%), bowel (*n* = 345; 70.1%), and vitality/hormonal function (*n* = 223; 45.5%) domains at baseline (see Additional file 1: Fig. S3). Generally, responses were maintained for most items during radiotherapy treatment; while some decreased at study visit 2, they recovered to baseline scores at visit 3. Only items related to sexual domain (7, 8, and 9) showed a greater number of problems and worsened after radiotherapy (see Additional file 1: Fig. S3).

### Construct validity of EPIC-16

Correlations between different EPIC-16 domains were modest (r < 0.50 for all Spearman’s correlations at baseline), indicating that the EPIC-16 domains are conceptually distinct and merit independent measure (see Additional file 1: Table S1).

### EPIC-16 responsiveness

**Fig. 4:**
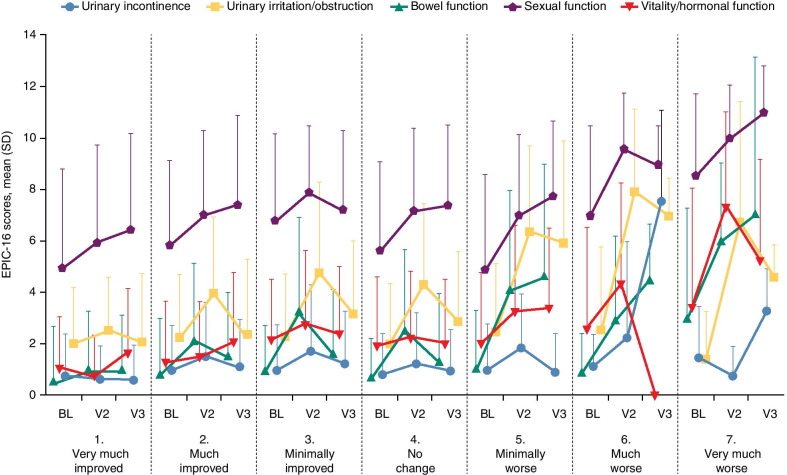
Mean EPIC-16 scores according to perceived changes in health status. *BL* Baseline, *V2* Visit 2 (end of radiotherapy), *V3* Visit 3 (90 days after the end of radiotherapy)

Compared to baseline, almost all EPIC-16 mean domain scores worsened at visit 2, regardless of the health-state change as perceived by the patient; similar results were reported at visit 3, with the exception of the urinary irritation/obstruction domain which improved (Fig. 4).

Patients who reported an improved health status at visit 2 compared with baseline generally had smaller effect sizes (i.e., 0.20 in magnitude) versus those who reported a worsened health status; similar results were also reported at visit 3 (Fig. 4).

### Correlation between EPIC-16 and UCLA-PCI

Overall, the EPIC-16 questionnaire scores showed strong correlations with the UCLA-PCI questionnaire domains at study visit 3, with Spearman’s correlations > 0.4 across all corresponding UCLA-PCI domains (Table 3). The urinary, bowel, and sexual function domains in the EPIC-16 questionnaire were most greatly correlated with respective domains in the UCLA-PCI questionnaire, e.g., the UCLA-PCI urinary function domain had a Spearman’s correlation coefficient with the EPIC-16 urinary incontinence domain of 0.713, the bowel domains with 0.579, and the sexual domains with 0.739.

**Table 3:** Correlation between EPIC-16 and UCLA-PCI scores at study visit 3

EPIC-16 domains	UCLA-PCI domains		
	Urinary function	Bowel function	Sexual function
Urinary incontinence	− .713	− .247	− .146
Urinary irritation/obstruction	− .468	− .301	− .065
Bowel	− .355	− .579	− .009
Sexual	− .152	− .094	− .739
Vitality/hormonal	− .254	− .314	− .210
Total score	− .509	− .429	− .443

All data are Spearman’s correlation coefficients

## Discussion

This observational, non-interventional, multicenter study in Spain measured the change in HRQoL in men with localized PCa undergoing radiotherapy using the EPIC-16 and UCLA-PCI questionnaires, and further validated the Spanish EPIC-16 for routine clinical practice. Overall, EPIC-16 scores worsened after radiotherapy in different HRQoL domains, regardless of patients’ perceptions of their health status, suggesting that patients did not perceive the change in functional domains as a global change in their health status. Similarly, the UCLA-PCI scores decreased with radiotherapy in both treatment groups (EBRT or BQT). However, 3 months after the end of radiotherapy, EBRT patients had recovered their scores in the urinary function, urinary bother, and bowel function domains. Conversely, BQT patients did not recover in urinary bother, bowel bother, and sexual function domains.

Similarly, in a long-term prospective HRQoL study in patients with localized PCa using EPIC-50, Ferrer, et al. reported that BQT treatment caused the least impact on HRQoL, except for moderate urinary irritative-obstructive symptoms, while sexual deterioration was observed for patients receiving EBRT [13].

The UCLA-PCI baseline values presented in this study were very similar to those reported by van de Poll-Franse, et al. in the Cancer of the Prostate Strategic Urologic Research Endeavor (CaPSURE) study, although the post-treatment scores of this study were smaller in magnitude compared to those of the CaPSURE study, which is likely due to the shorter period for the follow-up visits in this study (3 months) versus the 6–24-month follow-up period of CaPSURE [14].

Additionally, validation of the EPIC-16 questionnaire results obtained in this study was similar to results obtained by a number of EPIC-CP validation studies, especially those at study visit 2 [9, 15]. However, differences exist in the sexual and vitality/hormonal domains between these studies, which may be due to differences in use of concomitant hormone therapy [15, 16].

Even though the EPIC questionnaires were developed from UCLA-PCI [6, 7], both instruments were used in this study to further assess the validity of the Spanish version of the EPIC-16 questionnaire. The previous EPIC-CP study by Chang, et al. [9] was validated in a smaller study cohort (*N* = 307) compared to this study, with 175 treated and 132 untreated patients. Furthermore, the current study also included evaluation of longitudinal validity, in addition to correlational analysis between EPIC-16 and UCLA-PCI domains. Hence, the results presented here further add to these previously reported results for the English EPIC-CP.

The results of the current study further highlight the importance of using appropriate PRO instruments to aid physicians treating patients newly diagnosed with localized PCa, enabling them to decide appropriate treatment strategies, to consider their potential adverse effects, and to incorporate individual patient preferences [17–21].

This study showed that the Spanish version of the EPIC-16 questionnaire demonstrated sensitivity to detect both PCa treatment-related effects and sensitivity for clinical improvement after radiotherapy. Furthermore, it was also shown that EPIC-16 has strong discriminative properties and reliability, demonstrating its validity for use in clinical practice and clinical trials to evaluate the effect of interventions. The present study consolidates prior data reported by Balbotin, et al. in a small prospective series of 46 patients treated with radical prostatectomy, brachytherapy, and external beam radiotherapy [22]. The EPIC-16 domains showed correlations with the respective functional domains in the UCLA-PCI questionnaire. Although these results show that both questionnaires are highly correlated in the urinary, bowel, and sexual function domains, they do not measure exactly the same information and are therefore complementary.

In future, the validated Spanish EPIC-16 could be utilized in a larger-scale analysis with more longitudinal components to further assess PROs comparing different types of radiation therapies, similar to what has been recently done for EPIC-26 by Nossiter, et al. [23].

A main limitation of this study was its observational design and lack of an adjusted analysis by potential confounder clinical and therapeutic factors. The use of neo-adjuvant and concomitant hormone therapy might impact data concerning sexual function [16]. Higher use of neo-adjuvant/androgen deprivation therapy in patients treated with EBRT versus those treated with BQT is a limitation for the interpretation of results and may have impacted differences observed in the sexual and hormonal/vitality domains, as reported previously [24]. Furthermore, despite several studies suggesting that neo-adjuvant/androgen deprivation therapy in addition to radiotherapy increased the incidence of urinary or rectal toxicity [25], other studies have not corroborated these findings [26, 27]. In addition, the smaller-than-planned sample for the BQT group affected the power of the hypothesis testing and resulted in large CIs and lack of statistical significance. It should be noted that the changes seen in BQT patients are not permanent; these patients were still on treatment due to the nature of the therapy. On the other hand, the main strength of the study is the inclusion of 516 patients from 41 radiation oncology departments in Spain, a highly representative sample of clinical practice in our country.

## Conclusions

In this observational study conducted in Spain, men with localized PCa undergoing radiotherapy reported worsened scores in different HRQoL domains of the EPIC-16 and UCLA-PCI questionnaires immediately after radiotherapy treatment; however, a strong tendency towards recovery was seen at the 3-month follow-up visit. Regardless, patients did not perceive a global HRQoL change. Validation of the Spanish version of the EPIC-16 demonstrated sensitivity, strong discriminative properties and reliability, and validity. This shortened questionnaire is therefore suitable for use in routine clinical practice in Spain to measure urological HRQoL in this population.

## Supplementary Information

**Additional file 1: Table S1**. Spearman’s correlation coefficients between EPIC-16 domains at baseline. **Fig. S1**. Mean baseline EPIC-16 and UCLA-PCI domain scores according to type of radiotherapy. **Fig. S2**. Evolution of overall urinary quality of life in the EPIC-16 questionnaire according to type of radiotherapy. **Fig. S3**. Evolution of responses in all items of the EPIC-16 questionnaire.

## Data Availability

Researchers may request access to anonymized participant-level data, trial-level data, and protocols from Astellas sponsored clinical trials at http://www.clinicalstudydatarequest.com. For the Astellas criteria on data sharing, see: https://clinicalstudydatarequest.com/Study-Sponsors/Study-Sponsors-Astellas.aspx.

## Abbreviations

BQT: Brachytherapy
CaPSURE: Cancer of the Prostate Strategic Urologic Research Endeavor
CI: Confidence interval
EBRT: External beam radiotherapy
EPIC: Expanded Prostate Cancer Index Composite
HRQoL: Health-related quality of life
ICC: Intraclass correlation coefficient
NRS: Numeric response scale
PCa: Prostate cancer
PRO: Patient-reported outcome
SD: Standard deviation
UCLA-PCI: University of California-Los Angeles Prostate Cancer Index
