# Supplementary material for: Health-related quality of life in men with localized prostate cancer treated with radiotherapy: validation of an abbreviated version of the Expanded Prostate Cancer Index Composite for Clinical Practice in Spain
Source: Health Qual Life Outcomes. 2021 Sep 25;19:223. doi: 10.1186/s12955-021-01856-z (PMC8466718; doi:10.1186/s12955-021-01856-z)
Supplement: Supplementary file 1 — Additional file 1: Table S1. Spearman’s correlation coefficients between EPIC-16 domains at baseline. Fig. S1. Mean baseline EPIC-16 and UCLA-PCI domain scores according to type of radiotherapy. Fig. S2. Evolution of overall urinary quality of life in the EPIC-16 questionnaire according to type of radiotherapy. Fig. S3. Evolution of responses in all items of the EPIC-16 questionnaire. [file 12955_2021_1856_MOESM1_ESM.docx]

**Additional material**

**List of investigators***

Meritxell Arenas, Anna Boladeras Inglada, Patricia Cabrera Roldán, Francisco Celada Álvarez, Elia del Cerro Peñalver, Eduardo Ferrer Albiach, Palmira Foro Arnalot, Tamara García Cañibano, Luis Alberto Glaria, David Gomez Gomez, Alfonso Gómez-Iturriaga, Carmen González San Segundo, Pedro Mª González Acosta, Mª Rosario Guerrero Tejada, Víctor Macías Hernández, Xavier Maldonado Pijoan, Asunción Hervás Morón, Josep Jové Teixidó, Alfonso Mariño Cotelo, Agustina Méndez Villamon, Moisés Mira Flores, Rodrigo Muelas Soria, Julia Luisa Muñoz García, Cristina Nuño Rodríguez, Jesús Olivera Vegas, Amalia Palacios Eito, José Pardo Masferrer, Pedro J. Prada Gómez, Daniel Rodríguez Domínguez, Juan Ignacio Rodríguez Melcón, Alvar Roselló, Pilar Samper Ots, Enrique Sánchez Aparicio, Gemma Sancho Pardo, Amalia Sotoca Ruiz, Begoña Taboada Valladares, Izaskun Valduvieco Ruiz, Jeannette Valero Albarrán, Berta Valls, Manuel Gonzalo Vázquez Masedo, Elena Villafranca Iturr, Irma Zapata Paz, Almudena Zapatero.

* Recruited patients into the study

**Psychometric validations of the Spanish version of the EPIC-16 questionnaire**

The psychometric properties of the EPIC-16 questionnaire were evaluated for feasibility, reliability, construct, and longitudinal validity and sensitivity to change for the total number of patients included in the study at baseline versus the follow-up visits. Feasibility was assessed by calculating the ceiling and floor effect, i.e., the number of patients who responded with the maximum and minimum scores, respectively. Reliability was assessed using internal consistency by calculating the Cronbach’s alpha coefficient, where ≥ .7 was expected, and test–retest reliability by calculating the intraclass correlation coefficient for the number of patients who stated that their health state was unchanged between baseline and visit 2. Construct validity was analyzed using a 5-factor analysis. Longitudinal validity was evaluated for changes observed, including seriousness, radiotherapy group, and the UCLA-PCI questionnaire domains, using bivariate tests corresponding to the characteristics of the variables analyzed. Sensitivity to change was assessed using the overall EPIC-16 scores according to the change in the patient’s perceived overall change in health status. The calculated effect size quantifies the size in difference between two time points, defined as small (< .2), moderate (~ .5), large (~ .8), very large (~ 1.2), or exceptionally large (> 2.0).

**Additional file 1: Table S1.** Spearman’s correlation coefficients between EPIC-16 domains at baseline

|  | EPIC-16 domains | | | | | |
| --- | --- | --- | --- | --- | --- | --- |
| EPIC-16 domains | Urinary incontinence | Urinary irritation/obstruction | Bowel function | Sexual function | Vitality/hormonal function | Total score |
| Urinary incontinence | 1.000 | .386 | .130 | .179 | .127 | .460 |
| Urinary irritation/obstruction | .386 | 1.000 | .236 | .229 | .374 | .653 |
| Bowel function | .130 | .236 | 1.000 | .149 | .330 | .432 |
| Sexual function | .179 | .229 | .149 | 1.000 | .270 | .753 |
| Vitality/hormonal function | .127 | .374 | .330 | .270 | 1.000 | .615 |
| Total score | .460 | .653 | .432 | .753 | .615 | 1.000 |

**Additional file 1: Fig. S1** Mean baseline EPIC-16 (A) and UCLA-PCI (B) domain scores according to type of radiotherapy


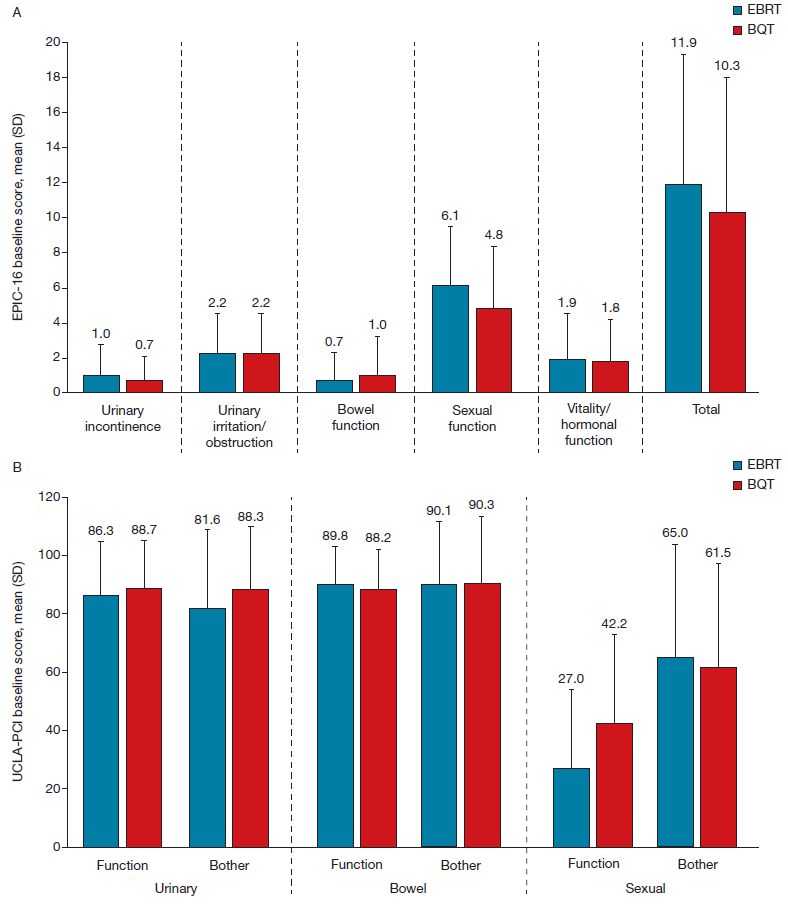


**Additional file 1: Fig. S2** Evolution of overall urinary quality of life in the EPIC-16 questionnaire according to type of radiotherapy


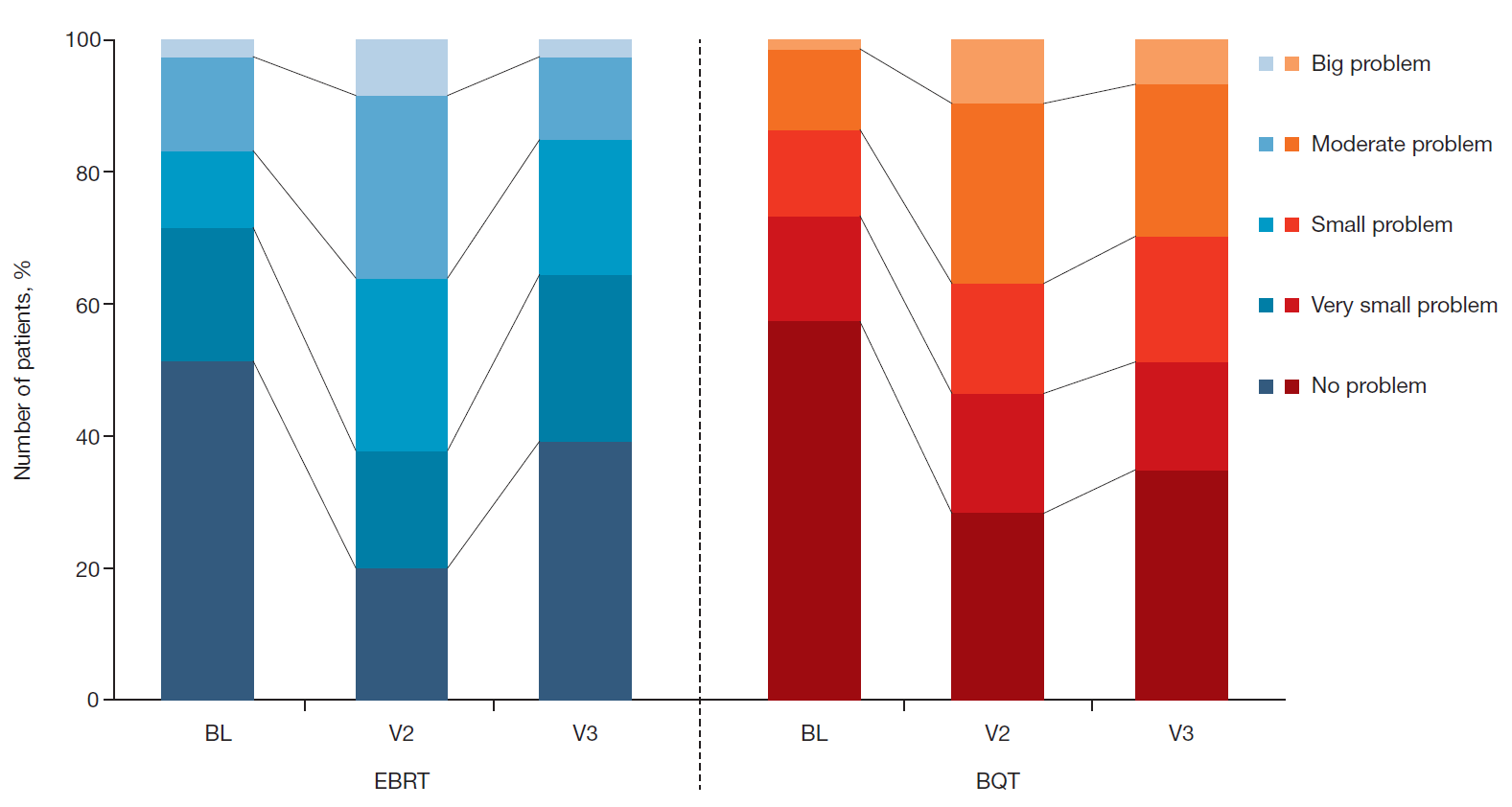


*BL* Baseline, *V2* Visit 2 (end of radiotherapy), *V3* Visit 3 (90 days after the end of radiotherapy)

**Additional file 1: Fig. S3** Evolution of responses in all items of the EPIC-16 questionnaire


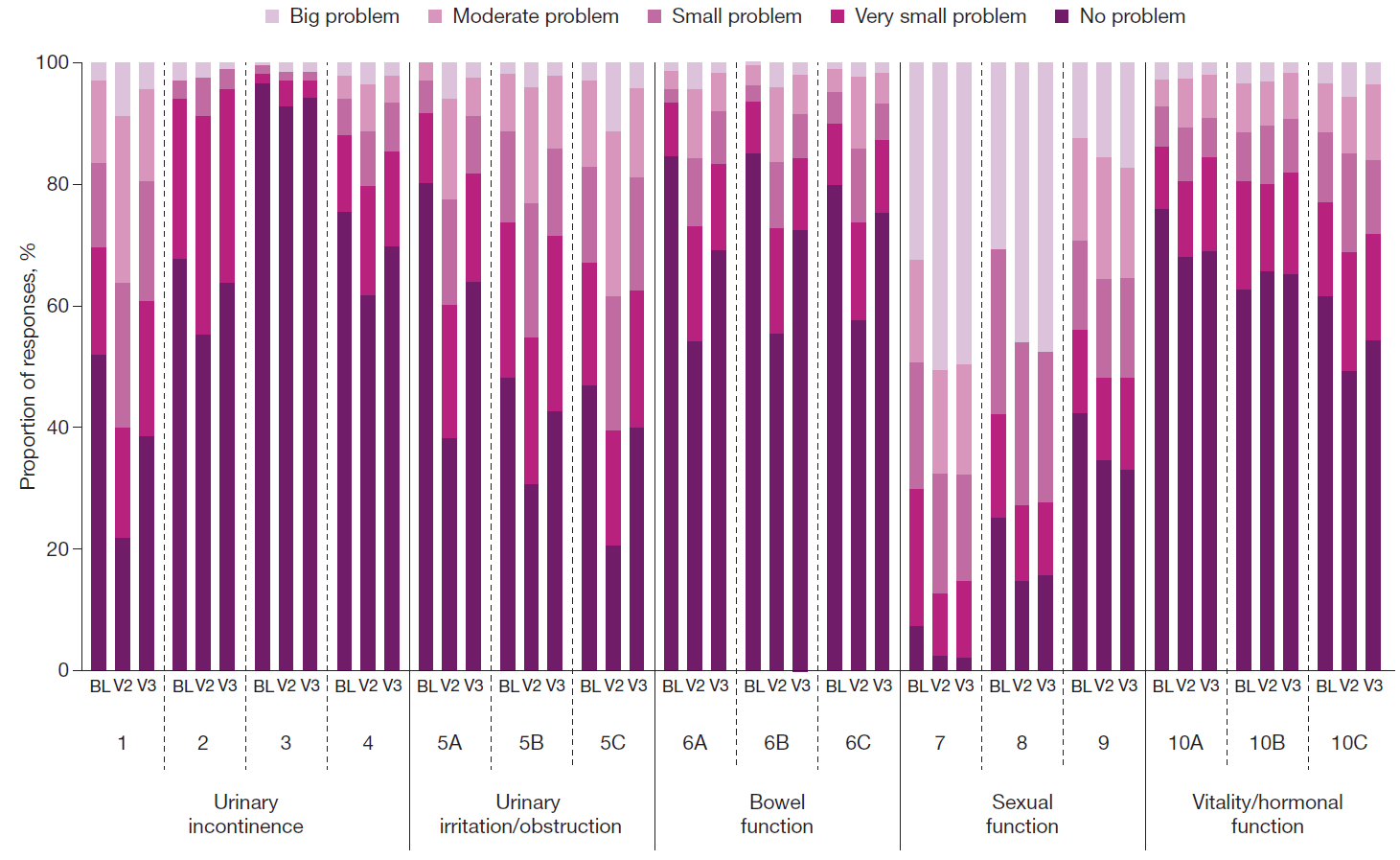


*BL* Baseline, *V2* Visit 2 (end of radiotherapy), *V3* Visit 3 (90 days after the end of radiotherapy)
